# Psychometric Properties of the Resilience Scale for Adolescents (READ) and Measurement Invariance Across Two Different German-Speaking Samples

**DOI:** 10.3389/fpsyg.2020.608677

**Published:** 2020-12-24

**Authors:** Clarissa Janousch, Frederick Anyan, Odin Hjemdal, Carmen Nadja Hirt

**Affiliations:** ^1^Institute for Research and Development, School of Education, University of Applied Sciences and Arts Northwestern Switzerland, Windisch, Switzerland; ^2^Department of Psychology, Norwegian University of Science and Technology, Trondheim, Norway; ^3^Institute Secondary Level I and II, School of Education, University of Applied Sciences and Arts Northwestern Switzerland, Windisch, Switzerland

**Keywords:** Germany, Switzerland, protective factors, factor analysis, resilience, Resilience Scale for Adolescents, validation

## Abstract

The Resilience Scale for Adolescents (READ) is a highly rated scale for measuring protective factors of resilience. Even though the READ has been validated in several different cultural samples, no studies have validated the READ across samples in German from Switzerland and Germany. The purpose of this study was to explore the construct validity of the German READ version in two samples from two different countries and to test the measurement invariance between those two samples. A German sample (*n* = 321, *M* = 12.74, *SD* = 0.77) and a German-speaking Swiss sample (*n* = 349, *M* = 12.67, *SD* = 0.69) of seventh graders completed the READ, Hopkins Symptom Checklist (HSCL-25), Rosenberg Self-Esteem Scale (RSE), General Self-Efficacy Scale, and Satisfaction with Life Scale (SWL). The expected negative correlations between READ and HSCL-25 and the positive correlations between RSE, self-efficacy, and SWL were supported. Furthermore, the results of the measurement invariance demonstrated that the originally proposed five-dimensional structure is equal in the German and Swiss samples, and it can be assumed that the same construct was assessed by excluding one item. The five-factor, 27-item solution is a valid and reliable self-report measure of protective factors between two German-speaking samples.

## Introduction

According to the World Health Organization (WHO, 2019), half of all mental health conditions start by the age of 14, and 15% of all adolescents (aged 10–19) are affected by mental health disorders. However, most cases remain undetected and untreated, which might be problematic because extended mental health conditions can deteriorate physical and psychological health and limit opportunities to lead a fulfilling life as an adult (WHO, 2019).

Until the late 1970s, the concept of pathogenesis, pioneered and developed by Williamson and Pearse (1980), was predominant in medicine and health-care systems (Antonovsky, 1996). This approach aims to determine the origin and cause of certain diseases and retrospectively try to avoid, manage, or terminate the disorder (Becker et al., 2010). In contrast, Antonovsky’s (1979) salutogenesis approach focused on preventing mental disorders. Psychological ill-being should be absent, and the presence of psychological well-being is needed. Therefore, the main aim is to maintain or even improve health and psychological well-being by fostering health-promoting factors (Langeland and Vinje, 2017). Such factors are salutogenic factors, resilience factors, positive mental health, a supportive social system, and life satisfaction, which act in protective health-promoting processes (Bibi et al., 2020). Thus, it is crucial to determine, measure, foster, and strengthen protective factors and understand the risks associated with the physical, social, and economic aspects and vulnerability (Proag, 2014). These protective factors underlie positive psychological development (Masten, 2011) and help individuals resist in times of risk and adversity or balance the risk to which they are exposed (Rutter, 1985, 2012).

In this regard, resilience has gained interest in research and applied practice over the last three decades because it is an essential source of subjective well-being (Snyder et al., 2011; Estrada et al., 2016). Resilient resources can buffer the negative consequences of facing adversity and maintain (Connor and Davidson, 2003) or even improve psychological and physical health (Ryff and Singer, 2000). In scientific literature, various definitions of resilience can be found. Its complexity has been widely acknowledged, which has led to no universal operational definition of resilience (Luthar et al., 2000; Kaplan, 2002; Masten, 2007). However, five key concepts of resilience can be identified in the empirical literature (Aburn et al., 2016). The first concept focuses on a certain ‘ability’ of individuals to *rise above to overcome adversities* (e.g., Rutter, 1990; Garmezy, 1991; Bonanno, 2004). While certain definitions state a *positive adaptation and adjustment* is linked to resilience (e.g., Rutter, 1987; Werner and Smith, 1992; Luthar, 2006), a key concept which has been cited by many studies (e.g., Dowrick et al., 2008; Canvin et al., 2009; Janssen, 2011) is Masten’s ‘*ordinary magic.*’ Resilience is a common phenomenon which is an interplay between an individual’s strengths and the support of family, friends and the external social environment (Masten, 2001). Measuring the absence or low incidence of mental health despite ongoing adversity is a method used by several researchers. Thus, g*ood mental health* despite significant adversity is considered a proxy for resilience (e.g., Masten et al., 1999; Bonanno et al., 2002; Connor and Davidson, 2003). Finally, the fifth key concept is the *ability to bounce back* from adversity, based on the word’s Latin origin ‘resiliere’ meaning to jump back (e.g., Werner, 1990; Luthar et al., 2000; Luthar, 2006). Notwithstanding the difference in the key concepts, they all include a stable trajectory and outcome of individual and communal healthy functioning after a highly adverse event (Southwick et al., 2014). Furthermore, because it is a dynamic adaptation and development process, it changes throughout a lifetime and is multidimensional (Rutter, 2000; Scheithauer et al., 2000; Opp et al., 2008; Wustmann, 2008). Masten (2014, 6) defines resilience broadly as “the capacity of a dynamic system to adapt successfully to disturbances that threaten system function, viability, or development.” According to Hjemdal et al. (2006, 84), resilience can be defined as “the protective factors, processes, and mechanisms that contribute to a good outcome despite experiences with stressors shown to carry significant risks for developing psychopathology.”

Even though some exposure to adversity is important for adolescents’ growth and development of resilience (Masten, 2014), it is well established that adolescence is perceived as a crucial stage in human life and development and thus, being exposed to severe stress, trauma and adversities can lead to a set of negative neurodevelopmental, psychological and physiological outcomes (Putnam, 2006; Brady and Back, 2012). These adolescents are at risk of suffering from posttraumatic stress disorder (PTSD), depression, anxiety, disruptive behaviors, and substance abuse (McLaughlin and Lambert, 1970). Especially in times of the coronavirus disease 2019 (COVID-19) there is high prevalence of psychological distress in adolescents (e.g., Havnen et al., 2020; Prime et al., 2020; Ran et al., 2020).

Three overarching categories of resilience are generally accepted by researchers in adolescence: *positive individual factors*, *social family support*, and characteristics of the *supportive environment outside the family* (Garmezy, 1983; Werner, 1989, 1993; Rutter, 1990; Werner and Smith, 1992; Hjemdal, 2007). Firstly, *positive individual factors* include self-system variables, such as positive self-concept, motivation, internal locus of control, sense of coherence, engaging temperament, sociability, and low emotionality (Werner, 2000, 118). Additionally, Olsson et al. (2003, 5) mention robust neurobiology, intelligence, and communication skills. Secondly, perceived *social family support*, parental warmth, encouragement, and assistance, as well as close relationships with parents, and cohesion and care within the family are crucial (Masten et al., 1988; Olsson et al., 2003; Leidy et al., 2010). Thirdly, the broader social environment, such as the neighborhood, work, and school, plays a significant role in the last category: *supportive environment outside the family*. Adolescents who use social support systems effectively and form strong bonds with peers and teachers are considered more resilient. It is vital to have nurturing and competent individuals who are supportive and prosocial in order to overcome adversities (Masten, 2009), because it is associated with several psychological and behavioral mechanism, including motivation, feelings of being understood, increased self-esteem and the use of active coping strategies (Southwick et al., 2016).

The lack of a clear definition and the interpretation of resilience as a conceptual umbrella for factors that can modify the impact of adversities (Hjemdal et al., 2006) has led to the development of a number of scales (Windle et al., 2011). However, there are several significant critiques regarding the wide variety of (indirect) measures assessing resilience. First, most of these instruments have been used to assess adults’ resilience, and only a few scales are available for young children and adolescents. For example, Youth Resiliency: Assessing Developmental Strengths (Donnon et al., 2003; Donnon and Hammond, 2007), Adolescent Resilience Scale (Oshio et al., 2003), The Resilience Scale (Wagnild and Young, 1993), Psychological Resilience (Windle et al., 2008), and Ego Resiliency (Bromley et al., 2006) can be applied for children and adolescents. Even though the READ (Hjemdal et al., 2006) has originally been adapted from the Resilience Scale for Adults (RSA; Friborg et al., 2003), the wording and response format has been simplified, shortened and validated and is therefore considered adequate for the use in adolescent samples (see *Development and Validation of the READ*). Secondly, most of these instruments do not cover all three introduced overarching categories of resilience (*positive individual factors*, *social family support*, and characteristics of the *supportive environment outside the family*), and they differ significantly and focus mainly on individual factors. Among the just mentioned scales, only the Youth Resiliency: Assessing Developmental Strengths (YR: ADS) measures dimensions apart from individual factors, such as family, community, and peers. Nevertheless, this scale includes 10 dimensions and 94 items, which might be too lengthy and extensive for certain research applications. Thirdly, some of these scales are in the early stages of development and all need further validation (Windle et al., 2011). Thus, these aspects hamper the possibilities of making valid comparisons. However, the READ (Hjemdal et al., 2006) is the only direct measure of resilience for adolescents that incorporates all three overarching protective factors (*positive individual factors*, *social family support*, *supportive environment outside the family;* Kelly et al., 2017), in a five-factor scale (*Personal Competence, Social Competence, Structured Style, Family Cohesion, Social Resources*). It has been validated in different countries and with different samples (von Soest et al., 2010; Stratta et al., 2012; Ruvalcaba-Romero et al., 2014; Kelly et al., 2017; Moksnes and Haugan, 2018; Askeland et al., 2019; Pérez-Fuentes et al., 2020).

The present study explores the validity and reliability of the READ (Hjemdal et al., 2006), a measure of protective factors of resilience, in two independent German-speaking samples in Germany and Switzerland. Even though the scale has been previously validated and thus is a valid and reliable instrument in several countries and languages, it has not been validated in a German-speaking sample so far. Furthermore, the samples differ in sizes, ages, and linguistic and cultural backgrounds. Therefore, following a literature review, the validation process and results will be outlined, and finally, the use of the READ in practice and empirical applications will be addressed.

### Development and Validation of the READ

The READ (Hjemdal et al., 2006) was developed in Norway and adapted from the Resilience Scale for Adults (RSA; Friborg et al., 2003). Initially, the RSA consisted of 41 items, which were simplified for the READ by changing two critical aspects. First, the semantic differential-type response format was changed to a five-point Likert format. Secondly, the wording was simplified, and phrases were only positively formulated to improve interpretation and completion of the survey. Furthermore, two items were excluded because they were considered irrelevant. With the remaining 39 items, a structural equation *post hoc* modeling and a confirmatory factor analysis (CFA) were conducted, with a sample of 421 adolescents aged 13–15 years, resulting in a 28-item, five-factor structure with acceptable model fit indices. The five factors of the READ comprised all three generally accepted higher categories of resilience, and the READ scale was organized into five factors, which are represented in the six factors of the RSA. The first overarching factor, *positive individual factors*, is represented by the three dimensions, *Personal Competence*, *Social Competence*, and *Structured Style*. The second and third overarching factors, *social family support* and *supportive environment outside the family* are covered by the subscales *Family Cohesion* and *Social Resources*.

The *Personal Competence* factor includes factors that measure several individual aspects, such as self-esteem, self-efficacy, ability to uphold daily routines, or the ability to plan and organize. The *Social Competence* factor focuses mainly on excellent communication skills and flexibility in social matters. The *Structured Style* factor measures the preference of an individual to plan and structure their daily routines. The *Family Cohesion* factor measures family support and the family’s attitude toward life in times of adversity. Lastly, the *Social Resources* factor measures perceived access to a social environment outside the family, such a relatives and friends, and the availability of their support (Hjemdal et al., 2006). The READ has been validated in five countries (Norway, Italy, Mexico, Ireland, and Spain), which do not all support the original factor structure and have raised questions about the factor-item patterns. So far, seven further validations of the READ (von Soest et al., 2010; Stratta et al., 2012; Ruvalcaba-Romero et al., 2014; Kelly et al., 2017; Moksnes and Haugan, 2018; Askeland et al., 2019; Pérez-Fuentes et al., 2020) have been published in English speaking journals, showing that the five-factor 28-item solution might be problematic. The number of factors differ, as do the number of items and which item(s) should be removed.

von Soest et al. (2010) conducted exploratory factor analysis, using a sample of 6,723 adolescents, aged 18–20, and based in Norway, which supported a five-factor solution. Nevertheless, the following confirmatory factor analysis supported the findings of the exploratory factor analysis for further improvement. Therefore, five items were removed, and an acceptable fit for all factors and the overall model was obtained. Stratta et al. (2012) collected data from 446 students in their final year of high school (18–20 years) in Italy, and found a four-dimensional structure by using principal component analyses and confirmatory factor analyses. *Personal Competence* and *Structured Style* factors were combined. However, cross-loadings on 17 of 28 items were > 0.40, indicating the items were not contributing to measuring the construct itself. Similarly to Stratta et al. (2012), Ruvalcaba-Romero et al. (2014) conducted a principal component analysis but removed six items in their five-factor solution. The composed five-factor structure differed from the original structure in two dimensions. *Social Competence*, *Family Cohesion*, *Social Resources* remained largely the same, whereas *Personal Competence* comprised only four instead of eight items, measuring mainly self-confidence. Furthermore, the remaining *Personal Competence* items and a few items of the *Structured Style* factor were combined in a new factor called Goal–Orientation. This data was collected from a sample of 840 adolescents (12–17 years) in Mexico.

Just like von Soest et al. (2010) and Ruvalcaba-Romero et al. (2014), Moksnes and Haugan (2018) excluded several items for their final solution by using confirmatory factor analysis. However, they kept the original five-factor structure, and the 20-item version showed good model fit indices. The sample was based on 1,183 adolescents, aged 13–18 years, based in Norway.

Criticizing the use of confirmatory factor analyses and exploratory factor analyses, Askeland et al. (2019) conducted, aside from the just mentioned analyses, an exploratory structural equation modeling with a sample of 9,596 students, aged 16–19 years, from Norway. The exploratory factor analysis model, as well as the exploratory structural equation model, showed good model indices for a 28-item, five-factor solution, whereas the results of the confirmatory factor analysis for the same model were poor. Finally, Pérez-Fuentes et al. (2020) tested the Spanish 22-item, five-factor version of Ruvalcaba-Romero et al. (2014) with 317 students (13–18 years) in Spain by using a principal component analysis followed by confirmatory factor analysis. Results indicated that the originally proposed structure by Ruvalcaba-Romero et al. (2014) was the best model and therefore showed good fit indices. However, they tested the 22-item version instead of the original five-factor, 28-item structure.

There is only one study so far that could confirm the original structure by using a confirmatory factor analysis without any modifications (Kelly et al., 2017). The study was conducted in Ireland, and 6,030 students, aged 12–18 years, participated. Unlike other previous studies (e.g., von Soest et al., 2010), Kelly et al. (2017) used a confirmatory approach and a common factor analysis framework in validating the READ, overcoming some of the methodological limitations in previous studies. For example, the exploratory approach (e.g., EFA) is used when the latent structure of a measure is unknown or when a confirmatory approach fails to reproduce an initial measurement model. It is unclear why EFA preceded CFA in validating a known latent structure of the READ in the studies conducted by von Soest et al. (2010); Stratta et al. (2012), and Ruvalcaba-Romero et al. (2014). As the PCA is not a common factor analytic framework, it fails to account for measurement unreliability (Brown, 2015); thus, previous studies using the PCA (Stratta et al., 2012; Ruvalcaba-Romero et al., 2014; Pérez-Fuentes et al., 2020) did not overcome the limitations of measurement unreliability. Three studies investigated measurement invariance across gender (Kelly et al., 2017; Moksnes and Haugan, 2018; Askeland et al., 2019) resulting in acceptable model fit for both genders. However, only Askeland et al. (2019) showed metric and partial scalar invariance for their newly built 5-factor solution by conducting an ESEM.

The construct validity of the READ has been supported by negative correlations with, on the one hand, depression (Hjemdal et al., 2006; Hjemdal, 2007; Skrove et al., 2013; Moksnes and Haugan, 2018) and anxiety (e.g., von Soest et al., 2010; Hjemdal et al., 2011, 2015; Skrove et al., 2013). Both constructs have been well investigated and provide evidence for validity (McLaughlin and Lambert, 1970). On the other hand, self-esteem (e.g., Ruvalcaba-Romero et al., 2014; Kelly et al., 2017; Moksnes and Haugan, 2018), and self-efficacy (Sagone and Caroli, 2013), are well-known indicators for construct validity. However, satisfaction with life has, to the authors’ knowledge, not been included in previous validation studies yet, but is according to Seligman (2002) positively connected to resilience, and thus, included in the present study.

The literature review has demonstrated several strengths in previous validations, such as sample sizes and age ranges. However, certain methodological weaknesses need to be addressed. There is a lack of correspondence between the results of the confirmatory factor analysis (CFA), exploratory factor analysis (EFA), and exploratory structure equation modeling (ESEM) because the factor analysis techniques may not be fully comparable (van Prooijen and van der Kloot, 2001). CFA is usually used for testing an instrument’s theoretical structure; thus, the latent structure is already known and the most widely used method for investigation factorial invariance (Byrne et al., 1989). Items are only allowed to load on one factor. EFA, which has been used as the most common approach to validate the READ scale, presupposes no structure, and the number of cross-loadings is not limited. This exploratory approach can also be used in cases of unsatisfactory confirmatory analyses. ESEM is a combination of the CFA and EFA approaches by allowing unrestricted estimations of all factor loadings (Fischer and Karl, 2019). According to Mai et al. (2018), ESEM might be a necessary method for large samples and substantive cross-loadings in the model, which cannot be ignored. Nevertheless, it remains unclear why exploratory approaches have been used to validate the READ in previous papers, and therefore, comparisons to previous studies should be made cautiously.

### Present Study

Previous validations have shown that there is not only a lack of methodological clarity but also a lack of a validated version of the READ in any German-speaking countries. German is the native language to more than 100 million people, mainly spoken in Central Europe (Germany, Austria, Switzerland, Lichtenstein, and some parts in Italy, Belgium, Luxembourg, and Poland). Even though these countries and regions share a common language, they differ in their cultural backgrounds and dialects (Krieger et al., 2020). Some words and phrases might not be understood or have a different meaning in another country/region. However, irrespective of the cultural, educational, or individual differences of these countries and regions, it is indispensable to have an adequately adapted questionnaire understandable for all German-speaking regions.

Therefore, this study examines the psychometric properties and the factorial validity of the German READ in a German and Swiss sample of adolescents in seventh grade by conducting a multi-group factor analysis (MGCFA).

Even though most studies have used exploratory approaches to validate the READ, Kelly et al. (2017) were able to replicate the original five-factor structure proposed by Hjemdal et al. (2006). Thus, we hypothesize that,

(i) the German version of the READ is a reliable and valid instrument, and the five-factor structure of the READ is consistent with the model proposed by the developers (Hjemdal et al., 2006) and the replication by Kelly et al. (2017).

Furthermore, Pérez-Fuentes et al. (2020) were able to replicate the five-factor structure proposed by Ruvalcaba-Romero et al. (2014), showing that even though the scale has been shortened, the structure replicates in two independent samples sharing a common language but having different cultures. Therefore, in this study, it was expected that,

(ii) not only form invariance but also metric invariance can be expected, indicating the equivalence of factor loadings across the German and Swiss samples.

However, the equivalence in residual variances was not expected or regarded as a requirement for a valid use of the READ within the German-speaking countries (Hjemdal et al., 2015).

Lastly, several studies (e.g., Hjemdal et al., 2006, 2011, 2015; Hjemdal, 2007; von Soest et al., 2010; Skrove et al., 2013; Moksnes and Haugan, 2018) have found that the READ correlates negatively with depression and anxiety, on the one hand. On the other hand, the READ correlates positively with self-esteem (e.g., Ruvalcaba-Romero et al., 2014; Kelly et al., 2017; Moksnes and Haugan, 2018), self-efficacy (Sagone and Caroli, 2013), and satisfaction with life (Seligman, 2002). This leads to the hypothesis that,

(iii) the READ correlates negatively with anxiety and depression but positively with self-esteem, self-efficacy, and satisfaction with life.

### Cross-Cultural Validations

Due to the rapid pace of globalization, the worldwide population diversity has increased and calls for a need to ensure that assessment tools function equivalently across contexts and that the same construct manifests similarly and, therefore, is measured similarly. Researchers and clinicians conduct more multinational and multicultural studies and have to adapt instruments for use in other languages and cultures (Beaton et al., 2000). However, the adaptation of longer scales is challenging when trying to maintain the meaning of the original measurement and keeping a relevant and comprehensible form (Sperber et al., 1994; Sousa and Rojjanasrirat, 2011). Cross-cultural or cross-national studies try to examine the validity of an instrument across cultures and nations. A specific hypothesis will not be tested; rather, the demonstration of equivalence (the absence of a construct, method, and item bias) will be explored (Matsumoto and van de Vijver, 2011).

However, these cross-cultural studies vary according to the contexts (Beaton et al., 2000), and even though the same language is used in a questionnaire, cultural backgrounds need to be taken into account. Therefore, two independent samples using the same language version of the questionnaire can help identify cultural differences in certain items for one version of a scale.

Substantive differences in the meaning of resilience and how resilience manifests across the two independent samples could restrict the generalizability of findings. This could be the result of differences in the scale locations or the starting points that different people use to scale their responses on the READ scale despite having the same values on the latent resilience construct. Measurement invariance examines whether the same construct has been measured in the same way across different people, contexts, and cultures (Meredith, 1993; Steenkamp and Baumgartner, 1998; Chen, 2007; Marsh et al., 2010; Millsap, 2011). Measurement invariance can pinpoint any sources of non-invariance across a hierarchy of levels, ranging from equal form to equal intercepts. These levels are required for comparing scale means across independent samples when differences exist in the way the construct has been measured or interpreted by independent samples (Milfont and Fischer, 2010).

## Materials and Methods

### Participants

This cross-sectional sample is based on the National Centres of Competence in Research (NCCR) project On the Move—The Migration-Mobility Nexus Overcoming Inequalities with Education—School and Resilience, funded by the Swiss National Science Foundation (SNSF). Six hundred and seventy seventh graders from lower secondary education classes (ISCED 2) in Germany and Switzerland completed the German-speaking web-based survey of risk and protective factors of mental health. The mean age of the random sample in Germany (*n* = 321), ranging from 11 to 16 years, was 12.74 (*SD* = 0.77), and 44.2% of the participants were female. Whereas, the mean age of the random sample in Switzerland (*n* = 349), ranging from 11 to 15 years, was 12.67 (*SD* = 0.69), and 45.6% of the participants were female (gender was not identified for seven participants).

### Procedure

The research has been conducted in accordance with the World Medical Association’s Declaration of Helsinki. The Ministry of Baden-Württemberg (Germany), the Cantonal Bureau for Education in the Cantons of Aargau, Basel-City, and Solothurn, and the Ethics Committee (for psychological and related research) of the Faculty of Arts and Social Sciences of the University of Zürich endorsed the data collection. First, the students’ questionnaires were translated into seven languages (Arabic, English, Farsi, French, German, Greek, and Turkish). A first translator translated the questionnaire into the target language, and a second independent translator checked the translation via retranslation. In the end, they decided on a consensus questionnaire. For the present study, only German-speaking questionnaires have been considered, as German is also the primary language in the three participating Swiss cantons. Afterward, schools in rural and urban areas in the Federal State Baden-Württemberg and the Cantons of Aargau, Basel-City, and Solothurn were asked to participate in the study. The headmaster of each school and the teachers approved participation in the survey. All parents and students received information letters explaining the procedure, and participation and data collection was voluntary, anonymous, and confidential. Participants were free to withdraw at any point in the study. Written informed consent to participate in the study was provided by the students and by their legal guardians. Students were asked to fill out the web-based questionnaires individually at their convenience on tablets. Teachers helped to administer the surveys during a regular school session of 90 min. Those not present while the study was carried out were asked to participate later.

### Measures

*Gender* and *age* were collected as single-item indicators for demographical information.

#### Resilience Scale for Adolescents

The READ (Hjemdal et al., 2006) is a 28-item self-report scale composed of only positively phrased items and a 5-point Likert-type response scale, ranging from 1 (*totally disagree)* to 5 (*totally agree)*. Higher scores indicate higher levels of resilience (Hjemdal et al., 2006). The scale with its five subscales (*Personal Competence, Social Competence, Structured Style, Family Cohesion, and Social Resources*) is a valid and reliable measurement for resilience.

#### Depression and Anxiety

The Hopkins Symptom Checklist (HSCL-25; Mattsson et al., 1969; Derogatis et al., 1974) is a self-report mental health screening questionnaire for measuring depression and anxiety. The scale has been used before for construct validity of the READ and includes 25 items (15 items about depression and 10 items about anxiety), originally derived from the 90-item Symptom Checklist (SCL-90; Derogatis and Savitz, 1999). It is a four-point Likert scale, ranging from 1 (*Not bothered)* to 4 (*Extremely bothered*), with a high internal consistency. One item was omitted (“Loss of sexual interest or pleasure”) due to the participants’ age range.

#### Self-Esteem

The Rosenberg Self-Esteem Scale (RSE; Rosenberg, 1965) measures global self-worth by using ten positively, as well as negatively, phrased items. The RSE allows adolescents to rate items on a four-point Likert scale, ranging from 1 (*Strongly disagree)* to 4 (*Strongly agree)*. Higher scores on the unidimensional high internal consistency scale (Cronbach’s alpha coefficients are usually above 0.80, and values above 0.90 have been reported) indicate a higher level of self-esteem (Heatherton and Wyland, 2003).

#### Self-Efficacy

To measure general self-efficacy, Schwarzer and Jerusalem’s (1995) scale has been used. With its good internal reliability, the 10-item psychometric scale assesses optimistic self-beliefs on a 4-point Likert scale, ranging from 1 (*Not at all true)* to 4 (*Exactly true)*. The total score of the scale, which is available in 33 languages, is calculated by finding the overall sum. The total score ranges between 10 and 40, indicating more self-efficacy with a higher score.

#### Satisfaction With Life

The Satisfaction with Life Scale (SWL; Pavot and Diener, 1993) is a brief self-report scale consisting of five items and a 7-point Likert response scale, ranging from 1 (*Strongly disagree)* to 7 (*Strongly agree*). A score of 20 represents a neutral point on the scale, whereas scores of 5–9 indicate extreme dissatisfaction, and scores of 31–35 indicate extreme satisfaction with life. According to Pavot and Diener (1993), the scale has high internal consistency and good test–retest correlations (0.84, 0.80 over a month interval).

### Statistical Analyses

For the confirmatory factor and measurement invariance analyses, Mplus version 8 (Muthén and Muthén, (1998-2017)) was used. First, confirmatory factor analysis was conducted, using robust maximum likelihood estimation with robust standard error procedures to account for non-normality and the five categories of the READ (Rhemtulla et al., 2012). The model fit was assessed using the Comparative Fit Index (CFI), the Tucker-Lewis Index (TLI), the Root Mean Square Error of Approximation (RMSEA), and the Standardized Root Mean Square Residual (SRMR). CFI and TLI values higher than 0.90 indicate an acceptable fit, whereas values greater than 0.95 correspond to an excellent fit (Hu and Bentler, 1999). According to Hu and Bentler (1999) and Bühner (2011), an RSMEA between 0.08 and 0.10 is considered acceptable. The same applies to the SRMR (Browne and Cudeck, 1992). Modification indices and, therefore, possible options for adjustment of the measurement model were inspected if the model fit was not acceptable.

Secondly, configural, metric, and scalar invariance for the CFA model across the two national samples were examined (Cieciuch and Davidov, 2016). Configural invariance, which also represents the baseline model, was tested first. It is satisfied when the latent structure is invariant across groups (in this case, across a German and Swiss sample), thus supporting the idea of an identical number of factors and a pattern of factor-item relations across the two groups (Brown, 2015). According to Putnick and Bornstein (2016), metric (weak) invariance is met when different groups respond to items in the same way, resulting in a comparable rating. This test can be conducted by constraining all factor loadings as equal across groups. Scalar (strong) invariance provides information about the comparison in latent constructs across groups by implying that participants “with the same value on the latent construct should have equal values on the observed variable” (Hong et al., 2003, 641). It is satisfied by constraining the intercepts of items to be equal across groups. However, it is rarely supported (Marsh et al., 2018).

The Chi square-test statistics have disadvantages due to the sensitivity of the sample size (Hayduk et al., 2007), and a compensatory test for measurement invariance for higher sample sizes was conducted (Cheung and Rensvold, 2002). According to Chen (2007) and Putnick and Bornstein (2016), a ΔCFI of ≤ 0.01 supplemented by an ΔRMSEA of ≤ 0.015 indicates invariance when testing weak invariance.

Thirdly, reliability and validity were evaluated by using IBM SPSS Statistics, version 24 (IBM Corp, 2016). Cronbach’s alpha and McDonald’s (1970) omega were computed, and convergent validity was assessed by correlating the five factors of the READ with the HSCL depression and anxiety dimensions, as well as the RSE, self-efficacy, and SWL, where certain patterns of correlation were expected. These correlation coefficients, retrieved from the two independent samples, were compared and tested to see whether a significant difference in the correlation of both cohorts could be found. Therefore, the test statistic *z*-value was calculated.

## Results

### Psychometric Characteristics of the READ

Table 1 presents the means, standard deviations, reliability estimates, and *z*-values of all measurement instruments and their subscales. The sum scores of the following scales and subscales were higher in Switzerland compared to the German sample: HSCL-25 Anxiety, RSE, READ total, Personal Competence, Social Competence, Social Competence, Structured Style, Family Cohesion, and Social Resources. Furthermore, both countries show mean scores in the HSCL-25 scale above the cut-off value for “caseness,” which is defined at 1.75 for the original English version (Winokur et al., 1984) and is widely used for several other languages (Mollica et al., 1987).

**TABLE 1:** Means, standard deviations, reliability, Pearson’s correlations between HSCL-25, RSE, self-efficacy, SWL, and READ scores for Germany (*N* = 321), and Switzerland (*N* = 349).

		*Mean*	*SD*	α 95% CI [LL, UL]	ω 95% CI [LL, UL]	1	2	3	4	5	6	7	8	9	10	11	12
(1) Age	Germany	12.74	0.77														
	Switzerland	12.67	0.69														
(2) HSCL total	Germany	1.85	0.60	0.94 [0.92, 0.95]	0.94 [0.92, 0.95]	–0.01											
	Switzerland	1.85	0.61	0.95 [0.93, 0.96]	0.94 [0.93, 0.96]	–0.03											
	Test statistic *z*					0.26											
(3) Depression	Germany	1.80	0.66	0.93 [0.91, 0.94]	0.93 [0.91, 0.94]	0.02	0.97**										
	Switzerland	1.78	0.67	0.93 [0.91, 0.94]	0.93 [0.91, 0.94]	–0.01	0.96**										
	Test statistic *z*					0.39	1.88*										
(4) Anxiety	Germany	1.93	0.60	0.83 [0.78, 0.85]	0.82 [0.78, 0.85]	–0.03	0.91**	0.77**									
	Switzerland	1.95	0.62	0.86 [0.82, 0.88]	0.86 [0.82, 0.88]	–0.05	0.91**	0.76**									
	Test statistic *z*					0.26	0.00	0.31									
(5) RSE	Germany	2.91	0.55	0.78 [0.73, 0.82]	0.75 [0.66, 0.81]	−0.14*	−0.53**	−0.56**	−0.41**								
	Switzerland	2.96	0.53	0.82 [0.77, 0.85]	0.80 [0.73, 0.85]	0.02	−0.57**	−0.60**	−0.44**								
	Test statistic *z*					−2.07*	0.74	0.78	0.47								
(6) Self-efficacy	Germany	2.92	0.58	0.89 [0.86, 0.92]	0.89 [0.85, 0.92]	–0.04	−0.22**	−0.22**	−0.17**	0.38**							
	Switzerland	2.88	0.53	0.88 [0.86, 0.90]	0.88 [0.86, 0.91]	0.16**	−0.34**	−0.34**	−0.31**	0.50**							
	Test statistic *z*					−2.59**	1.68*	1.68*	1.92*	−1.92*							
(7) SWL	Germany	5.07	1.38	0.78 [0.73, 0.82]	0.78 [0.74, 0.82]	−0.17**	−0.40**	−0.44**	−0.29**	0.53**	0.32**						
	Switzerland	4.96	1.26	0.80 [0.75, 0.83]	0.80 [0.75, 0.84]	–0.00	−0.53**	−0.56**	−0.41**	0.58**	0.47**						
	Test statistic *z*					−2.21*	2.14*	2.07*	1.76*	–0.93	−2.30*						
(8) READ total	Germany	4.05	0.54	0.90 [0.87, 0.92]	0.90 [0.86, 0.91]	–0.04	−0.39**	−0.42**	−0.32**	0.40**	0.50**	0.42**					
	Switzerland	4.09	0.47	0.89 [0.87, 0.91]	0.89 [0.86, 0.91]	0.03	−0.46**	−0.48**	−0.37**	0.51**	0.54**	0.59**					
	Test statistic *z*					–0.90	1.10	0.97	0.73	−1.79*	–0.71	−3.0*					
(9) Personal competence	Germany	3.80	0.65	0.70 [0.62, 0.77]	0.70 [0.60, 0.77]	–0.05	−0.40**	−0.37**	−0.34**	0.41**	0.53**	0.42**	0.87**				
	Switzerland	3.85	0.57	0.70 [0.65, 0.75]	0.71 [0.66, 0.73]	0.03	−0.41**	−0.41**	−0.36**	0.45**	0.53**	0.49**	0.84**				
	Test statistic *z*					–1.03	0.15	0.61	0.29	–0.63	0.00	–1.14	1.44				
(10) Social competence	Germany	3.94	0.71	0.66 [0.57, 0.73]	0.67 [0.56, 0.73]	–0.01	−0.20**	−0.18**	−0.20**	0.20**	0.34**	0.24**	0.75**	0.62**			
	Switzerland	4.04	0.63	0.68 [0.60, 0.73]	0.68 [0.59, 0.74]	0.08	−0.29**	−0.30**	−0.23**	0.37**	0.40**	0.32**	0.75**	0.56**			
	Test statistic *z*					–1.16	1.23	1.64	0.41	−2.39**	–0.90	–1.12	0.00	1.19			
(11) Structured style	Germany	3.59	0.81	0.60 [0.51, 0.67]	0.60 [0.51, 0.67]	0.07	−0.18**	−0.19**	−0.14*	0.21**	0.43**	0.20**	0.74**	0.64**	0.43**		
	Switzerland	3.66	0.70	0.57 [0.48, 0.64]	0.57 [0.47, 0.66]	0.03	−0.24**	−0.27**	−0.17**	0.25**	0.33**	0.41**	0.69**	0.56**	0.37**		
	Test statistic *z*					0.52	0.81	1.09	0.40	–0.54	1.51	−3.00**	1.32	1.61	0.92		
(12) Family cohesion	Germany	4.33	0.68	0.81 [0.76, 0.85]	0.81 [0.76, 0.85]	–0.04	−0.40**	−0.45**	−0.28**	0.40**	0.30**	0.42**	0.76**	0.53**	0.40**	0.47**	
	Switzerland	4.35	0.66	0.85 [0.81, 0.89]	0.86 [0.81, 0.88]	0.02	−0.46**	−0.49**	−0.35**	0.45**	0.40**	0.58**	0.77**	0.51**	0.43**	0.43**	
	Test statistic *z*					–0.77	0.95	0.66	1.00	–0.79	–1.47	−2.77**	–0.31	0.35	–0.47	0.65	
(13) Social resources	Germany	4.51	0.62	0.77 [0.69, 0.83]	0.77 [0.69, 0.82]	−0.17**	−0.30**	−0.28**	−0.27**	0.31**	0.29**	0.29**	0.73**	0.52**	0.49**	0.38**	0.49**
	Switzerland	4.51	0.57	0.79 [0.73, 0.84]	0.79 [0.73, 0.84]	–0.00	−0.28**	−0.28**	−0.24**	0.36**	0.35**	0.39**	0.70**	0.49**	0.48**	0.34**	0.43**
	Test statistic *z*					−2.21*	–0.28	0.00	–0.41	–0.73	–0.86	–1.46	0.79	0.52	0.17	0.59	0.98

*p < 0.05, **p < 0.01, α = Cronbach’s alpha, p < 0.01.

### Confirmatory Factor Analyses

The original 28-item, five-factor model of the READ was tested through a confirmatory factor analysis (CFA) across the two independent samples. The results showed that the fit indices yielded a fit with room for improvement in the German sample (*χ*^2^_(__340__)_ = 581.934, *p* < 0.001; RMSEA = 0.047 [90% CI = 0.041–0.054]; SRMR = 0.058 CFI = 0.876; TLI = 0.862), which was also the case for the Swiss sample (*χ*^2^_(__340__)_ = 595.875, *p* < 0.001; RMSEA = 0.047 [90% CI = 0.040–0.053]; SRMR = 0.060 CFI = 0.882; TLI = 0.868).

After freely estimating the covariance of two (Germany) and three (Switzerland) error terms to the items 28–14 and 16–11, and 7–1, 15–5, and 8–2, respectively, the fit indices still showed room for improvement. They have been freely estimated due to statistical reasons and similar wording content of the items. The modification indices further demonstrated one problematic item: item 4 (“*I am satisfied with my life up till now*”). Therefore, it was removed, which resulted in acceptable fit indices for both samples: Germany (*χ*^2^_(__312__)_ = 460.87, *p* < 0.001; RMSEA = 0.039 [90% CI = 0.031–0.046]; SRMR = 0.053 CFI = 0.918; TLI = 0.907), and Switzerland (*χ*^2^_(__311__)_ = 491.237, *p* < 0.001; RMSEA = 0.041 [90% CI = 0.034–0.048]; SRMR = 0.057 CFI = 0.912; TLI = 0.900). Hence, these results confirm the original five-factor structure of the READ by excluding one item, consequently leaving a 27-item solution (M1a and M1b in Table 2).

**TABLE 2:** Evaluations of multigroup measurement invariance across countries (MGCFA MI).

Model	Type of test	Compared with	χ^2^	*df*	*RMSEA*	*CFI*	*TLI*	*SRMR*	Δ*df*	Δ*CFI*	Δ*RMSEA*	Δ*SRMR*	Decision
M1a	Germany		460.873 *p* < 0.001	312	0.039[0.031, 0.046]	0.918	0.907	0.053					
M1b	Switzerland		491.237 *p* < 0.001	311	0.041[0.034, 0.048]	0.912	0.900	0.057					
M2	Configural invariance		951.887 *p* < 0.001	623	0.040[0.035, 0.045]	0.915	0.904	0.055					
M3	Metric invariance	M2	988.836 *p* < 0.001	645	0.040[0.035, 0.045]	0.911	0.903	0.067	22	−0.004	0.000	0.012	Accept
M4	Scalar invariance	M3	1033.580 *p* < 0.001	667	0.041[0.036, 0.045]	0.905	0.900	0.069	22	−0.006	0.001	0.002	Accept

*χ^2^, chi-square statistic; df, degrees of freedom; RMSEA, root mean square error of approximation, CFI, comparative fit index; TLI, Tucker Lewis index; SRMR, standardized root mean square error of approximation; Δ, change in statistical values.*

### Measurement Invariance

**FIGURE 1:**
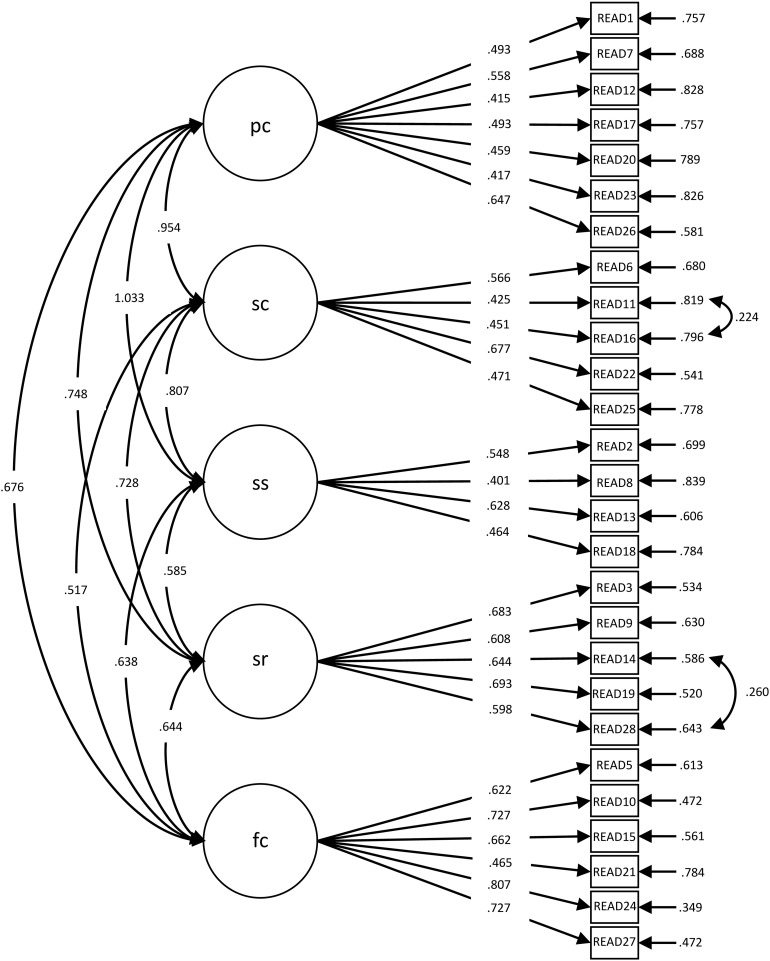
Factor loadings for the five-factor, 27-item model (Germany: *N* = 321). pc, personal competence; sc, social competence; ss, structured style; sr, social resources; fc, family cohesion.

**FIGURE 2:**
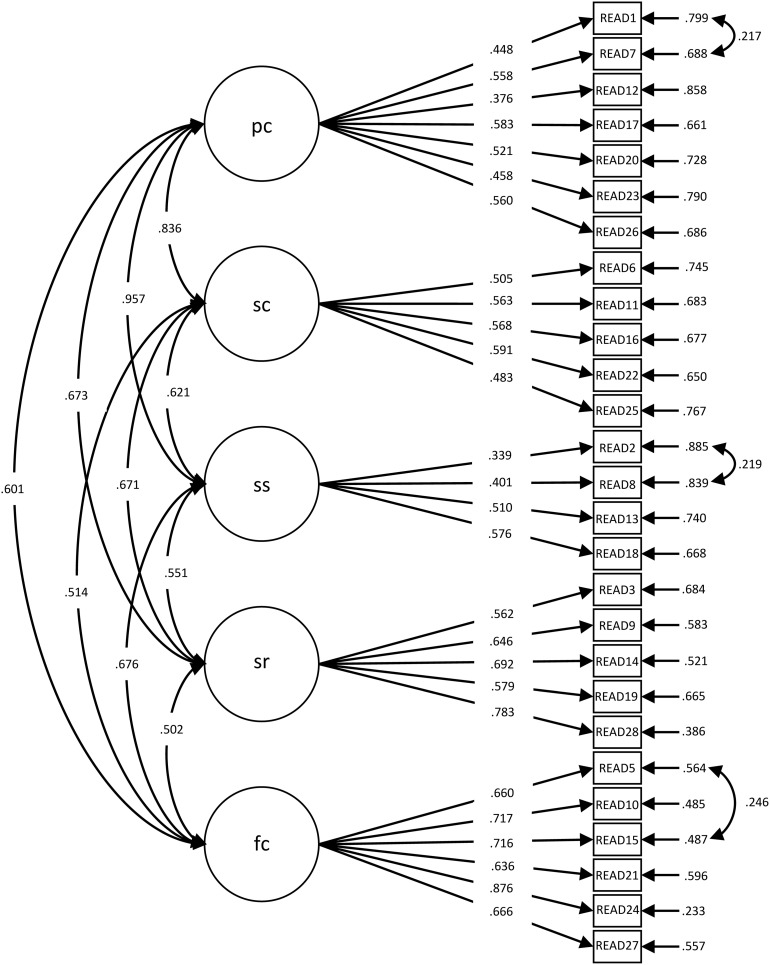
Factor loadings for the five-factor, 27-item model (Switzerland: *N* = 349). pc, personal competence; sc, social competence; ss, structured style; sr, social resources; fc, family cohesion.

The configural invariance (the baseline model, M2 in Table 2) was supported for the READ in both national samples. This model was compared to the model with constrained factor loadings equally across both groups (M3) to test what is considered together with the test for configural invariance to be the most important test of measurement invariance (Meredith, 1993; Meade and Lautenschlager, 2004; Vleeschouwer et al., 2014): metric invariance. The difference between the CFI values of the two models was below 0.01, and the differences between the RMSEA and SRMR values were below 0.015. Thus, the metric invariance was supported. The standardized factor loadings are presented in Table 3 and in Figures 1, 2. By constraining the intercepts to be the same across both groups, the *χ*^2^ value increased and resulted in a decrease in the fit of the scalar invariance model in comparison to the metric invariance model, as expected. However, the differences in the CFI, RMSEA, and SRMR values were below the cutoffs for rejecting invariance and, therefore, still supported scalar invariance.

**TABLE 3:** Standardized factor loadings in both countries.

Items	Germany (*N* = 321)					Switzerland (*N* = 349)				
	1	2	3	4	5	1	2	3	4	5
1 PC	0.49					0.45				
7 PC	0.56					0.56				
12 PC	0.42					0.38				
17 PC	0.49					0.58				
20 PC	0.46					0.52				
23 PC	0.42					0.46				
26 PC	0.65					0.56				
6 SC		0.57					0.51			
11 SC		0.43					0.56			
16 SC		0.45					0.57			
22 SC		0.68					0.59			
25 SC		0.47					0.48			
2 SS			0.55					0.34		
8 SS			0.40					0.40		
13 SS			0.63					0.51		
18 SS			0.46					0.58		
5 FC				0.62					0.66	
10 FC				0.73					0.72	
15 FC				0.66					0.72	
21 FC				0.47					0.64	
24 FC				0.81					0.88	
27 FC				0.73					0.67	
3 SR					0.68					0.56
9 SR					0.61					0.65
14 SR					0.64					0.69
19 SR					0.69					0.58
28 SR					0.60					0.78

*PC, personal competence; SC, social competence; SS, structured style; FC, family cohesion; SR, social resources.*

### Validity of the READ

To validate the READ, all five factors of the 27-item version were correlated with several psychological variables to investigate its convergent validity (see Table 1). As expected, the READ total score and subscales correlated significantly and negatively and of a low to moderate size with the HSCL-25 in both countries. The READ, as well as its subscales, correlated significantly and positively with the RSE (ranging from *r* = 0.20 to 0.51), self-efficacy (ranging from *r* = 0.29 to 0.54), and SWL (ranging from *r* = 0.20 to 0.59). The highest negative correlations were between HSCL-25 and the READ factors *Personal Competence* and *Family Cohesion*. The same subscale factors had the highest associations with RSE, self-efficacy, and SWL. In general, most of the correlations between all measurements were higher in the Swiss sample than in the German sample. However, testing for the significance of these correlations showed that only a few *z*-values were significant. The significant differences between the total READ scale and RSE (*z* = −1.79, *p* < 0.05), as well as the SWL (*z* = −3.0, *p* < 0.05), are higher in Switzerland than in Germany. The same applies for the subscale *Social Competence* and RSE (*z* = −2.39, *p* < 0.01), *Structured Style* and SWL (*z* = −3.00, *p* < 0.01), and *Family Cohesion* and SWL (*z* = −2.77, *p* < 0.01). The comparison of the correlations between the subscale *Social Resources* and age showed a significant *z*-value of −2.21 (*p* < 0.05), which was more negative for the German sample than for the Swiss sample.

## Discussion

The READ is an approved scale that continues to be used widely as a measure of protective factors of resilience (e.g., Windle et al., 2011). The primary aim of this first cross-cultural validation of the READ was to investigate the psychometric properties, measurement invariance, and construct validity. The main question was whether the READ is a valid scale to measure a similar latent construct of resilience across a German and a Swiss sample of seventh graders.

The first finding of the presented study was the support for the five-factor structure of the READ, even though one item had to be removed, thus supporting configural invariance. Item four, which has typically loaded poorly or inconsistently in previous studies of the READ, has been excluded several times (e.g., von Soest et al., 2010; Ruvalcaba-Romero et al., 2014; Moksnes and Haugan, 2018; Pérez-Fuentes et al., 2020). *“I am satisfied with my life up till now”* seems to be a problematic item, and its exclusion was justified statistically by showing high cross-loadings with the *Family Cohesion* factor and high modification indices. However, the item was designed to measure *Personal Competence* and cannot be statistically or clearly allocated to this factor. It can only be assumed that being satisfied with your own life might be strongly influenced by the family in this specific age range. The argument is founded on research by Szcześniak and Tułecka (2020), who demonstrated high positive correlations between satisfaction with life and family adaptability and cohesion. On the other hand, adolescents’ family stressors and life satisfaction correlate significantly and negatively (Chappel et al., 2014), showing that family influences an adolescent’s satisfaction with life. Therefore, item four seems to tap into this duality, which does not coincide with the classical test theory. Although the item may yield interesting information about this relation, it should be used with caution, particularly when exploring the factors in question. Future replicative studies with broader age-ranges are needed to determine if it would be better to leave it out for German or Swiss adolescent samples. Nonetheless, it appears that seventh graders from Germany and Switzerland conceptualize protective factors of resilience similarly, as reflected by the five factors.

The most important test of invariance, metric invariance, was also supported, showing that the factor loadings are equal for both groups. Thus, participants in Germany and Switzerland understand the items similarly and, therefore, interpret the wording of the items equally; their scores on the scale are comparable. Thus, it allowed simple regression analyses to predict comparable changes in criterion-related outcome variables across the two samples. Even scalar invariance was confirmed and therefore demonstrated that participants with the same value on the latent construct have identical values on the observed variable in both countries. These results show that the comparisons across the German and Swiss sample are meaningful and valid, direct mean comparisons are possible, and it can be assumed the same construct was assessed.

The internal consistency was supported by Cronbach’s alpha, which was 0.90 for the total READ score in the German sample and 0.89 in the Swiss sample but varied in both countries for the five READ factor subscales between 0.57 and 0.85. While the reliability of four subscales is satisfactory, the subscale *Structured Style* is rather low. This factor was the weakest one in previous studies (Hjemdal et al., 2006; von Soest et al., 2010; Kelly et al., 2017; Moksnes and Haugan, 2018). This alpha value could be caused by a low number of items (four items), poor interrelatedness between these items, or a heterogeneous construct (Tavakol and Dennick, 2011). Hence, this factor should be used with caution and be investigated further.

The construct validity of the READ was supported in the German, as well as in the Swiss sample, as the READ total score correlated significantly and negatively with the HSCL-25 total score and its subscales that measured anxiety and depression, and significantly and positively correlated with self-esteem, self-efficacy, and the newly integrated aspect satisfaction with life. These correlations were low to strong in size, with the expected directionality, and therefore supported construct validity. These findings are in accordance with previous studies. However, the significant differences in the correlation coefficients need to be further evaluated, as there are no studies available that have investigated similar differences. Hence, the significant differences could be related to higher levels of resilience and self-esteem among the Swiss sample, which could also mean that the Swiss sample may have relatively more access to resilience resources to boost their self-esteem. Thus, it is not surprising that the Swiss sample scored relatively higher on *Personal Competence* than the German samples because higher self-esteem contributes to higher personal competence. As mentioned, these findings are to be used with caution because more studies are needed for further evaluations.

Moreover, the READ is also relevant in practical and applied settings. The READ requires, compared to other resilience scales (e.g., Ego Resiliency by Bromley et al., 2006, including 102 items), a considerable length of time and thus might have lower rates of non-responses and missing data. Apart from only showing that the scale is a valid and reliable instrument to measure protective factors, providing test-retest information to indicate the instrument’s stability would be helpful. This would also give for example teachers or clinicians a possibility to use the scale in order to know an individual’s weaknesses and strengths in times of adversities and foster their resilience. Especially in times of Covid-19, where the mental health among adolescents could very likely decrease (Guessoum et al., 2020), knowing the availability of assets and resources could guide a framework for intervention (Windle et al., 2011).

Several limitations in this study need to be recognized. First, the sample consists of only seventh graders in one federal state of Germany and four cantons of Switzerland. We suggest that further measurement invariance analyses, ideally cross-cultural analyses, under different school levels and other regions, are needed to assess the generalizability of the READ scale. The study showed that seventh graders in the Federal State Baden-Württemberg and three cantons of Switzerland—Aargau, Basel-City, and Solothurn—understood the instrument in a similar fashion. On all measurement invariance levels, the findings of both samples did not differ significantly. Nonetheless, the samples have not been further investigated. Therefore, it remains unclear how these two samples differ according to their cultural, educational and individual backgrounds. Further studies could shed light on the comparability of a German and a Swiss sample.

Additionally, a further cross-cultural validation across two completely different samples (cultural and linguistic) would be of great benefit. As Germany and Switzerland are culturally similar (Kopper, 1993) and the language is almost the same, it raises the question of whether these results will be supported by a comparison between completely different cultures and two language versions, for example, between a Norwegian and Swiss sample. A study among 3,419 undergraduate students in 24 countries showed that language has an influence on response patterns. Half of all students in a country received an English-language questionnaire, whereas the other half filled out the same survey in their native language. Findings showed that cultural accommodations were present, and respondents altered their responses according to the culture of the language and, thus, the cultural values (Harzing, 2005). This strongly supports the idea of questionnaire translations and the idea trying to make several language versions available. To have these translations available (cross-national and cross-cultural), validations are essential.

Secondly, according to Hjemdal et al. (2006), a sample of individuals who have already dealt with long-term adversity and have overcome these adversities should have been chosen. Whether the students have dealt with such adversity has not been examined in detail. Even though, the mean values for the HSCL-25 total in both samples were above the cut-off value of *M* = 1.75 (Winokur et al., 1984): in the German sample *M* = 1.85 (*SD* = 0.60), and in the Swiss sample *M* = 1.85 (*SD* = 0.61). In addition, the sum scores of the subscales indicated “caseness,” and no specific sample facing adversity was investigated. Therefore, it would be interesting to have a closer look at a specific subsample with HSCL-25 values above 1.75 to examine the psychometric properties and factorial validity of a sample truly facing adversities. Furthermore, adolescents could have been categorized into depress/non-depressed or anxious/non-anxious adolescents based on the HSCL-25 to verify the difference in the READ scores among these groups. Also finding out to see if resilience as measured through the READ negatively predicts the total score at the HSCL-25 over and above self-esteem and self-efficacy could be a further way of showing incremental validity of the READ.

Thirdly, self-report measures always bear the risk of social desirability, especially when carrying out a study in the classroom (Althubaiti, 2016). To reduce this bias, students filled out the questionnaires separately in class on tablets, without seeing their peers’ answers. Furthermore, if there is a significant bias, the findings would differ for the two samples, or it can be assumed that the bias is similar for each sample. Finally, the cross-sectional nature of the present design must be noted. It is suggested that future research examines the consistency of the findings (test–retest reliability). However, the present study is the first cross-cultural validation study and the first validation study of the READ in adolescent samples from Germany and Switzerland. Further studies testing the reliability and validity in other countries and cultures in a cross-cultural design, focusing additionally on gender and age invariance, would be of great benefit to the READ.

## Conclusion

In conclusion, the first cross-cultural validation of the READ with samples from Germany and Switzerland provides further evidence on the psychometric properties of the READ as a valuable tool for the assessment of protective factors of resilience. The five-factor, 27-item model resulted in a good fit for the German and Swiss samples and has the potential for future research. Participants from both countries understood and interpreted these items in a similar and comparable fashion, which allows prospective studies to investigate adolescents’ mental health. However, modifications indicate a need for further investigations, whether the findings of the existing latent structure are replicable in different samples, countries, and cultures.

## Data Availability Statement

The datasets presented in this article are not readily available because the raw data supporting the conclusions of this article will be made available by the authors after 2023 when the project and dissertations will be completed. Requests to access the datasets should be directed to Clarissa Janousch, clarissa.janousch@fhnw.ch.

## Ethics Statement

The studies involving human participants were reviewed and approved by the Ministry of Baden-Württemberg (Germany), the Cantonal Bureau for Education in the Cantons of Aargau, Basel-City, and Solothurn; and Ethics Committee (for psychological and related research) of the Faculty of Arts and Social Sciences of the University of Zürich. Written informed consent to participate in this study was provided by the participants’ legal guardian/next of kin.

## Author Contributions

CJ collected the data, conducted the data analysis, wrote the first draft of the manuscript, and continuously revised and developed the final version based on the feedback received. FA, OH, and CH provided substantial feedback on the manuscript and data analysis. All authors contributed to the article and approved the submitted version.

## Conflict of Interest

The authors declare that the research was conducted in the absence of any commercial or financial relationships that could be construed as a potential conflict of interest.
